# RACIAL/ETHNIC DISPARITIES IN HOSPITAL READMISSION AND FREQUENT HOSPITALIZATIONS AMONG PEOPLE WITH DEMENTIA

**DOI:** 10.1093/geroni/igad104.0697

**Published:** 2023-12-21

**Authors:** Elham Mahmoudi

**Affiliations:** University of Michigan Medical School, Ann Arbor, Michigan, United States

## Abstract

People 65+ must choose between traditional Medicare (TM) and Medicare Advantage (MA). This study has two aims: (1) examine 30-day readmission and frequent hospitalizations in TM vs. MA; (2) examine racial/ethnic disparities in noted outcomes. We used a 20% random sample of TM and MA insurance claims (2018-2019). Participants included individuals 65+, with a diagnosis of Alzheimer’s disease and related dementia (ADRD), and two years of continuous enrollment in TM (n=129,177) or MA (n=119,130). Our explanatory variables included age, sex, race/ethnicity, comorbidities, frailty index, continuity of care index, and discharge location. Generalized linear models were applied. There were a higher percentage of Black (13.1% vs 8.2%) and Hispanic (11.9% vs. 7.0%) people in MA vs. TM (p <.001). MA enrollees had lower rates of chronic conditions and were less frail (31.4 vs. 33.6; p< .001), but a higher percentage of them had an annual wellness visit (38.8% vs. 32.3%; p <.001) than TM enrollees. Our matched and adjusted models show that TM increased the odds of readmission [OR=1.16 (95% CI: 1.06-1.26)] and 2+ hospitalizations [OR=1.25 (95% CI: 1.19-1.40)] compared to MA. Hispanic-White disparities in 30-day readmission [OR=1.23 (95% CI: 1.06-1.43)] and 2+ hospitalizations [1.42 (95% CI: 1/31-1.56)] were higher in TM vs. MA. Black people had higher readmission risks [OR=1.18 (95% CI:1.08-1.28)] and frequent hospitalizations [OR=1.26 (95% CI:1.19-1.33)] without any significant difference between MA and TM in Black-White disparities. MA vs. TM enrollees had lower risks of readmission and frequent hospitalizations. Moreover, MA lowered Hispanic-White disparities compared with TM.

